# Preeclampsia as an Independent and Major Risk Factor for Significant Postpartum Depression Symptomatology: Results from a Prospective Cohort Study

**DOI:** 10.3390/jcm15010395

**Published:** 2026-01-05

**Authors:** Larisa-Mihaela Holbanel, Adina Turcu-Stiolica, Daniela Gabriela Glavan, Sebastian Constantin Toma, Nicolae Cernea

**Affiliations:** 1 Doctoral School, University of Medicine and Pharmacy of Craiova, 200349 Craiova, Romania; dr.larisamihaela@gmail.com; 2 Department of Pharmacoeconomics, University of Medicine and Pharmacy of Craiova, 200349 Craiova, Romania; 3 Psychiatry Department, University of Medicine and Pharmacy of Craiova, 200349 Craiova, Romania; daniela.glavan@umfcv.ro; 4 General Surgery Department, Dr. Constantin Andreoiu, Ploiesti County Emergency Hospital, 100097 Ploiesti, Romania; 5 Department of Obstetrics-Gynecology, University of Medicine and Pharmacy of Craiova, 200349 Craiova, Romania

**Keywords:** preeclampsia, postpartum, depression, mental health

## Abstract

**Background/Objectives**: Preeclampsia is a severe hypertensive disorder that has been linked to increased maternal psychiatric morbidity. However, existing literature remains inconsistent regarding whether this association is independent of underlying medical co-morbidities such as chronic hypertension and diabetes. Our objective was to precisely evaluate the Adjusted Odds Ratio (AOR) of developing Postpartum Depression symptomatology (probable PPD) following a diagnosis of preeclampsia in a prospectively tracked cohort, controlling for essential confounders. **Methods**: This prospective cohort study included 180 women (33 in the Preeclampsia group, 147 in the Normotensive reference group), with stringent exclusion of women with prior psychiatric history to reduce confounding. PPD was assessed postpartum using the Edinburgh Postnatal Depression Scale (EPDS ≥ 13 cutoff). Multivariable logistic regression was employed to calculate the AOR, adjusting for maternal age, chronic hypertension, and prepregnancy diabetes. **Results**: The multivariable analysis demonstrated a highly significant and independent association between the primary exposure and the outcome. Preeclampsia was associated with 12.7-fold increased odds of developing PPD (AOR: 12.7; 95% CI: 5.1–31.7; *p* < 0.001). In contrast, none of the included confounders—chronic hypertension (AOR: 1.96, *p* = 0.182), prepregnancy diabetes (AOR: 1.8, *p* = 0.372), or age (AOR: 0.99, *p* = 0.759)—showed a statistically significant independent association with PPD risk. The model achieved strong explanatory power (Nagelkerke R^2^ = 0.327; Omnibus Test *p* < 0.001). **Conclusions**: Preeclampsia represents a powerful and independent determinant of the risk for significant PPD symptomatology, substantially increasing the adjusted odds of the condition. These findings mandate that women with a history of preeclampsia be designated a high-risk group and receive immediate, mandatory, and intensified postpartum mental health surveillance and preferential access to specialized psychological support.

## 1. Introduction

Preeclampsia is a severe hypertensive disorder of pregnancy, affecting 2–8% of pregnancies worldwide, and remains one of the leading causes of maternal and perinatal morbidity and mortality [1,2]. Initially regarded as a condition confined to gestation, preeclampsia is now recognized as a systemic disorder with long-term vascular and inflammatory sequelae, predisposing women to an increased risk of cardiovascular and metabolic diseases later in life [3,4,5].

Postpartum depression (PPD) represents a significant public health issue, affecting 10–20% of mothers within the first year after delivery [6,7]. The disorder not only compromises maternal well-being but also negatively influences the mother–infant relationship, the child’s emotional and cognitive development, and family functioning as a whole [8,9]. Despite the wide-ranging impact, PPD continues to be unrecognized and inadequately treated, particularly in settings with limited healthcare resources [10].

Although the long-term cardiovascular sequelae of preeclampsia are well established, its potential implications for maternal mental health remain less clearly defined. Several observational studies have suggested that women with a history of preeclampsia are at increased risk of developing postpartum depression (PPD) [11,12]. Nevertheless, other investigations have failed to demonstrate an independent association once adjustments were made for relevant confounders, including preexisting psychiatric conditions, psychosocial challenges of the perinatal period, and sociodemographic characteristics [13,14]. The inconsistency of these findings underscores both the complexity of the relationship and the need for further prospective research to determine whether preeclampsia constitutes an independent risk factor for PPD, or whether it primarily acts as an effect modifier in the context of broader psychosocial and biological vulnerabilities.

The biological plausibility of an association between preeclampsia and postpartum depression is supported by several interrelated mechanisms.

Preeclampsia is marked by systemic inflammation and endothelial dysfunction, processes that overlap with the inflammatory pathways implicated in the neurobiological models of depression [15,16]. Both conditions have also been linked to dysregulation of the hypothalamic–pituitary–adrenal axis, suggesting a common stress-related mechanism [17]. The etiology of postpartum depression is fundamentally linked to the rapid withdrawal of progesterone and estrogen, which triggers neurobiological fluctuations in susceptible women [18]. In the context of preeclampsia, this vulnerability is likely exacerbated by chronic dysregulation of the Hypothalamic–Pituitary–Adrenal (HPA) axis. Preeclampsia is characterized by abnormally high levels of placental-derived Corticotropin-Releasing Hormone (CRH), which leads to a state of chronic hypercortisolism followed by a precipitous “crash” in HPA axis activity after delivery of the placenta [19,20]. We propose that this chronic physiological stressor, combined with the pro-inflammatory milieu of preeclampsia, reduces the neuroplastic threshold of the maternal brain.

Furthermore, shared vascular risk factors, including cerebrovascular alterations, may contribute to the observed relationship [21]. Beyond these physiological links, the psychological burden of experiencing severe preeclampsia, preterm delivery, or neonatal complications may independently heighten susceptibility to postpartum depression [22].

The primary objective of this study was to determine whether preeclampsia constitutes an independent risk factor for PPD in a prospective cohort of women, after adjusting for sociodemographic, obstetric, and psychiatric confounders.

As secondary objectives, we explored the influence of additional maternal and perinatal variables on the association between preeclampsia and PPD such as sociodemographic factors (maternal age, education, marital status, employment), obstetric and perinatal factors (parity, gestational complications such as diabetes and hypertension), and postpartum clinical factors (postpartum blood pressure, maternal BMI).

By incorporating these variables, the study aimed not only to evaluate the independent effect of preeclampsia on PPD, but also to identify potential modifiers and mediators of this relationship.

## 2. Materials and Methods

We conducted a prospective cohort study located in Emergency County Hospital of Craiova, Romania, between 20 July 2024, and 30 June 2025. Ethical approval was granted by the Ethics Committee of the University of Medicine and Pharmacy of Craiova (Approval no. 196/18 July 2024), and we obtained written informed consent from all participants included.

Women eligible for inclusion were postpartum patients aged ≥18 years, with delivery after 28 weeks of gestation, and willingness to complete the Edinburgh Postnatal Depression Scale (EPDS) six weeks after delivery [23]. The exclusion of deliveries occurring 28 weeks before gestation (i.e., extreme prematurity) was a deliberate methodological choice. Deliveries at or before 24 weeks are highly associated with unique and severe levels of psychosocial stress due to periviability issues. By setting the exclusion boundary at 28 weeks, we aimed to isolate the independent effect of the preeclampsia disease process on postpartum depressive symptomatology, minimizing the potential for extreme prematurity to act as a singular, overwhelming psychosocial confounder that could obscure the primary association under investigation. The study population included women who either maintained normal blood pressure throughout pregnancy or received a diagnosis of preeclampsia. Preeclampsia diagnoses were established by specialist medical staff following the criteria set by the International Society for the Study of Hypertension in Pregnancy (ISSHP), which notably covers non-proteinuria preeclampsia and incorporates fetal growth restriction as a potential diagnostic feature [1]. Two groups were formed: Normotensive Group (systolic blood pressure < 140 mmHg and diastolic blood pressure < 90 mmHg) and Preeclampsia Group (hypertension after 20 weeks associated with proteinuria or evidence of maternal organ dysfunction).

The study employed the validated Romanian version of the EPDS, utilizing the established cut-point of 13 to screen for antenatal depressive symptoms [24]. The EPDS is a 10-item self-report questionnaire assessing symptoms experienced over the preceding seven days by pregnant individuals. Notably, the original Scottish developers of the instrument suggest that a score exceeding 10 may indicate possible depression, while scores above 13 warrant a referral for formal clinical assessment [23]. Cronbach’s alpha value of the questionnaire was computed and the value was 0.88.

Clinical and demographic data (maternal age, education level, obstetric history, medical history, and presence of hypertensive disorders) were collected from medical records.

The primary outcome was the presence of postpartum depressive symptomatology (probable PPD), evaluated six weeks postpartum using the EPDS questionnaire. A cut-off score ≥ 13 was used to indicate a high risk of PPD, maximizing combined sensitivity and specificity [25]. It is explicitly noted that the EPDS is a screening instrument and does not provide a definitive clinical diagnosis of Major Depressive Disorder (MDD). Therefore, our findings are interpreted as the risk of exhibiting significant depressive symptoms requiring clinical follow-up, not a confirmed psychiatric diagnosis.

Data analysis was undertaken in R packages (version 4.1, R Foundation for Statistical Computing, Vienna, Austria). Descriptive statistics were performed. Given the prospective cohort design with two defined groups (Preeclampsia and Normotensive) and the aim to adjust for confounders, we first compared baseline characteristics between the two groups. We used χ^2^ test (or Fisher’s exact test) for categorical variables (e.g., marital status, employment, PPD presence) and independent samples *t*-test (or Mann–Whitney *U* test for non-normal data) for continuous variables (e.g., maternal age, BMI, postpartum blood pressure).

The model will statistically predict the odds of PPD based on the preeclampsia status and the confounders. The general form of logistic regression will be:

$$\ln\left(\frac{P\left(PPD\right)}{1-P\left(PPD\right)}\right)=\beta_{0}+\beta_{1}(Preeclampsia)+\sum_{i=2}^{k}\beta_{i}({Confounder}_{i})$$

where is the logit of the odds of having PPD and *β*_1_ is the coefficient for the primary exposure (*Preeclampsia*).

## 3. Results

### 3.1. Characteristics of the Patients

**Figure 1:**
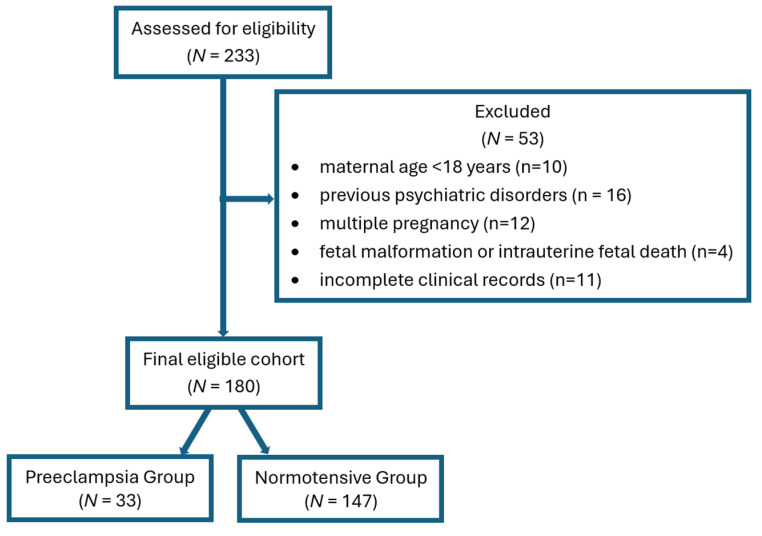
STROBE flow diagram.

For the study group, 233 women were initially evaluated, of whom 53 were excluded: maternal age < 18 years (*n* = 10), multiple pregnancy (*n* = 12), fetal malformation or intrauterine fetal death (*n* = 4), previous psychiatric disorders (*n* = 16, depression, anxiety, bipolar disorder, medication use: antidepressants, anxiolytics, mood stabilizers, psychotropic drugs), and incomplete clinical records (*n* = 11). Finally, 180 women were included in the study group analysis, from which 33 had preeclampsia. In conclusion, two groups (33 patients in Preeclampsia Group and 147 in Normotensive Group) were included for comparison of PPD risk. The selection process is shown in the STROBE flow diagram (Figure 1).

The baseline sociodemographic characteristics are summarized in Table 1. The mean age of the participants was slightly lower in the Preeclampsia group (28.39 ± 7.02 years) compared to the Normotensive group (30.66 ± 7.4 years), although this difference did not reach statistical significance (*p* = 0.098). Regarding educational attainment, the majority of women in both groups had achieved a university degree (61.2% in the Normotensive group vs. 54.5% in the Preeclampsia group), with no significant disparity observed (*p* = 0.556). Similarly, there were no significant differences between the cohorts regarding marital status, with 75.8% of women in the Preeclampsia group being married compared to 69.4% in the control group (*p* = 0.532). The distribution of residence (urban vs. rural) was also comparable (*p* = 0.681). Pre-pregnancy Body Mass Index (BMI) was virtually identical between the groups (27.46 ± 3.79 vs. 27.89 ± 4.37 kg/m^2^, *p* = 0.716), indicating that obesity rates were similar at baseline.

Obstetric history revealed that nulliparity was slightly more common in the Preeclampsia group (57.6%) than in the Normotensive group (55.1%), though this was not statistically significant (*p* = 0.848). However, significant differences emerged in maternal medical history (as shown in Table 1). A history of chronic hypertension was notably more prevalent in the Preeclampsia group (33.3%) compared to the Normotensive group (17.7%), showing a strong trend toward significance (*p* = 0.057). Furthermore, pre-pregnancy diabetes was significantly more frequent among women who developed preeclampsia (21.2%) compared to controls (7.5%) (*p* = 0.026), identifying it as a key risk factor in this cohort. A family history of preeclampsia was reported by 27.3% of the exposed group versus 19.7% of controls (*p* = 0.350).

As expected, clinical markers of the hypertensive disorder were markedly different. The Preeclampsia group exhibited significantly higher mean systolic blood pressure (138.45 ± 14.24 mmHg vs. 123.70 ± 12.43 mmHg; *p* < 0.0001) and diastolic blood pressure (85.85 ± 10.74 mmHg vs. 77.09 ± 10.00 mmHg; *p* < 0.0001) compared to normotensive women.

Regarding the primary outcome, scores on the EPDS were significantly elevated in the Preeclampsia group. The mean EPDS score was 13.48 ± 3.83, compared to 7.51 ± 3.93 in the Normotensive group (*p* < 0.0001). Consequently, the prevalence of PPD (defined as EPDS ≥ 13) was strikingly higher in women with preeclampsia (60.6%) than in the normotensive controls (9.5%) (*p* < 0.0001).

### 3.2. Model Building for PPD Symptomatology

**Figure 2:**
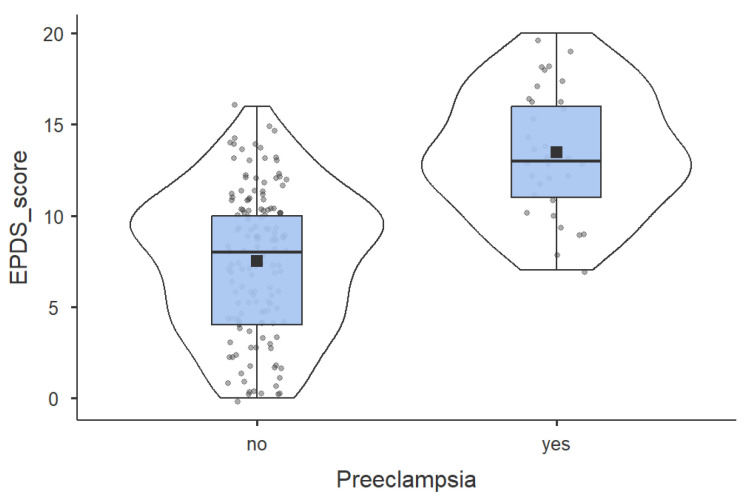
Comparation of EPDS score between Preeclampsia group and Normotensive group.

The mean EPDS score in Preeclampsia group was 13.48 ± 3.83 above the common cutoff for PPD (≥13), and 7.51 ± 3.93 in the Normotensive group (*p* < 0.0001), as shown in Figure 2. This suggests a very strong initial association, which will be the basis of our significant COR and AORs in the adjusted model of predicting the outcome of significant depressive symptomatology.

After analyzing the CORs in Table 2, we selected the optimal variables for AOR model, sticking to the maximum of 4 variables to prevent overfitting, given the 33 PPD cases. The final multivariable logistic regression model, which sought to establish the independent effect of preeclampsia on the risk of PPD, demonstrated a highly significant and robust association between the primary exposure (preeclampsia) and the outcome (PPD). After controlling for established and potential maternal confounders—namely age, prepregnancy diabetes, and chronic hypertension—women who had experienced preeclampsia showed significantly increased odds of developing PPD compared to normotensive women (AOR = 12.737; 95% CI: 5.113–31.726; *p* < 0.001). This result strongly confirms the hypothesis that preeclampsia represents a major independent risk factor for maternal mental health morbidity in the postpartum period, far surpassing the influence of common co-morbidities. Furthermore, while both prepregnancy diabetes (AOR = 1.803, *p* = 0.372) and chronic hypertension (AOR = 1.963, *p* = 0.182) showed AORs greater than 1, suggesting a tendency toward increased risk, neither reached statistical significance in this adjusted model.

The predictive validity of the multivariable logistic regression model was strongly established by its fit statistics. The Omnibus Test of Model Coefficients was highly significant (χ^2^(4) = 40.831, *p* < 0.001), confirming that the independent variables—Preeclampsia status, age, chronic hypertension, and prepregnancy diabetes—collectively contributed meaningfully to predicting the outcome of Postpartum Depression (PPD). Furthermore, the model demonstrated a moderate-to-strong explanatory power, with the Nagelkerke R Square indicating that the set of predictors accounted for 32.7% of the variability in PPD status. In terms of predictive accuracy, the model achieved a high Overall Percentage Correct of 86.1%. Crucially, while the model excelled at identifying the absence of PPD (correctly classifying 92.5% of PPD-absent cases), its sensitivity for detecting PPD was moderate, correctly identifying 20 out of 34 PPD cases, yielding a correct classification rate of 58.8%. This performance suggests the model is effective for epidemiological risk stratification but acknowledges that nearly half of true PPD cases are driven by unmeasured factors beyond the core clinical and demographic variables included in the final analysis.

The equation of the model predicting the odds of PPD is:

$$\ln\left(\frac{P\left(PPD=1\right)}{1-P\left(PPD=1\right)}\right)=-2.2+2.5\left(Preeclampsia\right)+0.7\left(Hypertension\right)+0.6\left(Diabetes\right)-0.01\left(Age\right)$$

For the binary variables (Hypertension, Diabetes, Preeclampsia), the term is calculated by multiplying the coefficient by 1 if the condition is present, and by 0 if the condition is absent (since the absence of the condition is the reference category).

A post hoc power analysis was conducted using G*Power (version 3.1.9.7) to evaluate the statistical power of the primary multivariable logistic regression model. Based on the total sample size of *N* = 180, a prevalence of significant postpartum depressive symptomatology of 9.5% in the normotensive (control) group, and the observed Adjusted Odds Ratio (AOR) of 12.74 for the preeclampsia group, the study achieved a post hoc statistical power (1 − *β*) exceeding 0.99 at an alpha level of 0.05. This indicates that despite the relatively small cohort, the substantial effect size observed (AOR > 13) provided more than sufficient statistical power to reliably detect the independent association between preeclampsia and postpartum depressive symptomatology.

## 4. Discussion

The primary objective of this prospective cohort study was to determine whether preeclampsia constitutes an independent risk factor for Postpartum Depression (PPD). Our principal finding is a highly significant and robust association: women diagnosed with preeclampsia faced a 12.7-fold increased adjusted odds (AOR: 12.737; 95% CI: 5.1–31.7) of developing PPD compared to normotensive women, after controlling for age, chronic hypertension, and prepregnancy diabetes. Recent studies have reported similar associations, indicating that women affected by preeclampsia have two- to four-fold higher odds of developing PPD within the first 6–12 weeks postpartum [26,27,28]. This result not only confirms the clinical suspicion of a link but elevates preeclampsia to a major, independent predictor of severe maternal mental health morbidity.

Several biological mechanisms may explain this relationship. Preeclampsia is characterized by systemic endothelial dysfunction, upregulation of inflammatory cytokines (e.g., IL-6, TNF-α), oxidative stress, and alterations in cerebral microvascular perfusion [29]. Recent neurobiological investigations suggest that such changes may alter neurotransmitter pathways implicated in mood regulation, including serotonergic and dopaminergic signaling [30]. When these physiological disturbances occur concurrently with abrupt postpartum hormonal shifts and psychosocial stressors, vulnerability to mood disorders may be substantially increased [31].

Clinically, these findings emphasize the need to view preeclampsia not solely as an obstetric complication, but also as a condition with important mental health implications. Although routine PPD screening is recommended for postpartum women, evidence suggests that women with preeclampsia should be identified as a high-risk group who require earlier and repeated mental health screening and targeted support during the first 6 weeks postpartum [28,32].

The magnitude of this finding is noteworthy, suggesting that the experience of preeclampsia is a far more powerful driver of PPD risk than underlying chronic conditions. In our adjusted model, neither prepregnancy diabetes nor chronic hypertension—both sharing inflammatory and vascular pathways with depression—reached statistical significance. This emphasizes the acuity and severity of the preeclampsia insult itself, rather than the preexisting medical environment, as the critical factor.

The biological plausibility for this association is strong and multifold. Preeclampsia is characterized by widespread endothelial dysfunction and systemic inflammation. Chronic inflammatory states are well-established as key mechanisms in the neurobiological model of depression, often disrupting neurotransmitter function and neurotrophic factor expression. Furthermore, the severe psychological burden of the preeclampsia syndrome—which often involves emergent delivery, prolonged maternal or neonatal intensive care stays, and feelings of guilt or failure—may synergize with these biological changes [33]. The subsequent and necessary detachment from the infant due to hospitalization likely disrupts early bonding, which is a known vulnerability factor for PPD. It is probable that the biological stress, combined with the psychosocial trauma, creates a “perfect storm” for PPD development [34].

Our findings resolve the inconsistencies noted in previous observational studies [13,14]. While some studies failed to find an independent link after adjustment, our use of a highly curated cohort, rigorous exclusion of pre-existing psychiatric conditions, and careful statistical modeling allowed us to isolate the strong, independent effect of the preeclampsia diagnosis. The high AOR observed in our cohort supports the most recent meta-analyses suggesting preeclampsia is not only a risk factor for development of depression, but it is also associated with higher severity of depressive symptoms [35]. This difference may stem from the sensitivity of our PPD screening and the clear stratification of our exposure groups.

A key strength of this investigation is its prospective cohort design, which enabled the rigorous establishment of temporal precedence, and the stringent exclusion of women with any prior psychiatric history or psychotropic medication use. and the stringent exclusion of women with any prior psychiatric history or psychotropic medication use. This approach ensures that the observed high prevalence of PPD is overwhelmingly due to new-onset episodes related to the preeclampsia syndrome, minimizing confounding by pre-existing mental health vulnerability. Foremost, the relatively small sample size (*N* = 180) and the resulting low number of PPD events (*n* = 33) introduced a constraint on the number of covariates in our multivariable model. While the AOR was highly significant, the wide confidence interval (95% CI: 5.113–31.726) reflects this statistical imprecision and suggests caution is needed when generalizing the precise magnitude of the risk to the broader population. Secondly, the model could not adjust for crucial psychosocial risk factors for PPD—such as pre-pregnancy relationship stress, financial hardship, social support, and the duration of maternal–infant separation (e.g., NICU stay). Specifically, the trauma associated with preterm birth and subsequent admission of the infant to a Neonatal Intensive Care Unit (NICU)—factors frequently associated with preeclampsia—are known to increase the risk of depression and anxiety [36]. As these factors are known PPD determinants, their absence may lead to residual confounding, potentially inflating the observed AOR. Thirdly, PPD diagnosis was based on the widely used EPDS screening cutoff (≥ 13), not a structured clinical interview, which may introduce minor classification bias. Finally, while we excluded women taking psychotropics, we did not perform a sub-analysis on the specific class of antihypertensive medications used in the preeclampsia group, acknowledging the potential for certain centrally acting drugs to influence mood. Despite these limitations, the robust magnitude and independence of the preeclampsia association remain a critical finding.

The compelling magnitude of our finding—that preeclampsia is an independent risk factor for a 12.7-fold increase in the odds of PPD—provides a strong and immediate mandate for changes in clinical screening and management. Women with a documented history of preeclampsia must be universally designated a high-risk group for PPD, necessitating a shift from opportunistic to mandatory, standardized psychological surveillance in the postpartum period. This screening should be integrated into both obstetric and primary care follow-up and persist beyond the typical 6-week window, acknowledging the often-delayed onset of PPD. From a public health perspective, these women require preferential access to perinatal mental health resources. Future research should prioritize mechanistic clarity: specifically, employing advanced models to test for mediation by key sequelae (e.g., the duration of neonatal intensive care unit stay, severe maternal anxiety, or persistent inflammatory markers). Disentangling these biological and psychological mediators is the next essential step toward developing precision preventative strategies, such as trauma-informed interventions for the birth experience or early immune-modulating therapies to reduce the long-term mental health burden.

## 5. Conclusions

Our research underscores the disproportionate impact of the preeclampsia event itself, rather than the underlying chronic medical conditions, as the primary driver of PPD risk in this cohort. This finding strongly supports the need for targeted, intensive psychological screening and supportive interventions for all women with a history of preeclampsia, extending well beyond the initial postpartum period.

## Figures and Tables

**Table 1:** Characteristics of included patients.

Characteristics	Normotensive Group *N* = 147			Preeclampsia Group *N* = 33			*p*-Value
	*N*	%	Mean ± SD and Median (IQR)	*N*	%	Mean ± SD and Median (IQR)	
Age			30.66 ± 7.4 32 (24–37)			28.39 ± 7.02 27 (22–34.5)	0.098 ^b^
Highest education							0.556 ^c^
- University degree	57	38.8		15	45.5		
- Secondary school	90	61.2		18	54.5		
Parity							0.848 ^c^
- multiparous	66	44.9		14	42.4		
- primiparous	81	55.1		19	57.6		
Environment							0.681 ^c^
- Urban	100	68		24	72.7		
- Rural	47	32		9	27.3		
Marital status							0.532 ^c^
- Married	102	69.4		25	75.8		
- Not married	45	30.6		8	24.2		
BMI prepregnancy			27.46 ± 3.79 27.5 (25.1–29.5)			27.89 ± 4.37 27.5 (24.7–31.1)	0.716 ^a^
Systolic blood pressure (mmHg)			123.7 ± 12.43 121 (114–128)			138.45 ± 14.24 139 (127–148.5)	<0.0001 ^b^
Diastolic blood pressure (mmHg)			77.09 ± 10.0 77 (69–83)			85.85 ± 10.74 86 (78.5–94)	<0.0001 ^b^
History_preeclampsia, yes	7	4.8		4	12.1		0.120 ^d^
FamilyHistory_preeclampsia, yes	29	19.7		9	27.3		0.350 ^c^
Chronic hypertension, yes	26	17.7		11	33.3		0.057 ^c^
Diabetes prepregnancy, yes	11	7.5		7	21.2		0.026 ^c^
EPDS			7.51 ± 3.93 8 (4–10)			13.48 ± 3.83 13 (11–16)	<0.0001 ^b^
PPD symptomatology	14	9.5		20	60.6		<0.0001 ^c^
Smoking, yes	27	18.4		2	6.1		0.115 ^d^
Alcohol, yes	6	4.1		0	0		0.594 ^d^

EPDS, Edinburgh Postnatal Depression Scale; PPD, postpartum depression; SD, Standard Deviation. ^a^ Independent Samples *t*-test. ^b^ Mann–Whitney U test; ^c^ Chi-square test. ^d^ Fisher’s exact test.

**Table 2:** Univariable and multivariable logistic regression.

Variable	COR (95% CI)	*p*-Value	AOR (95% CI)	*p*-Value
Preeclampsia (Ref: Normotensive)	14.6 (6.01–35.6)	<0.001	12.74 (5.1–31.7)	0.031
Age (per 1 year increase)	0.97 (0.92–1.02)	0.252	0.99 (0.93–1.05)	0.759
Education (Ref: High School)	1.06 (0.5–2.27)	0.876		
Marital status (Ref: unmarried)	1.77 (0.72–4.37)	0.208		
Environment (Ref: rural)	1.10 (0.49–2.5)	0.812		
Chronic hypertension (Ref: No)	2.64 (1.16–6.02)	0.018	1.96 (0.73–5.29)	0.182
Diabetes prepregnancy (Ref: No)	3.18 (1.13–8.95)	0.022	1.8 (0.49–6.58)	0.372
History preeclampsia (Ref: No)	1.67 (0.42–6.65)	0.437		
FamilyHistory_preeclampsia (Ref: No)	1.76 (0.75–4.09)	0.188		
Parity (Ref: primiparous)	0.98 (0.46–2.09)	0.966		
Smoking (Ref: No)	0.88 (0.31–2.49)	0.805		
Alcohol (Ref: No)	0.31 (0.02–5.7)	0.229		

COR (Crude Odds Ratio), AOR (Adjusted Odds Ratio).

## Data Availability

The data sets generated during and/or analyzed during the current study are available from the corresponding authors on reasonable request.

## References

[1] Brown M.A., Magee L.A., Kenny L.C., Karumanchi S.A., McCarthy F.P., Saito S., Hall D.R., Warren C.E., Adoyi G., Ishaku S. (2018). Hypertensive disorders of pregnancy: ISSHP classification, diagnosis, and management recommendations for international practice. Hypertension.

[2] Magee L.A., Brown M.A., Hall D.R., Gupte S., Hennessy A., Karumanchi S.A., Kenny L.C., McCarthy F., Myers J., Poon L.C. (2022). The 2021 International Society for the Study of Hypertension in Pregnancy classification, diagnosis & management recommendations for international practice. Pregnancy Hypertens.

[3] Bellamy L., Casas J.P., Hingorani A.D., Williams D.J. (2007). Pre-eclampsia and risk of cardiovascular disease and cancer in later life: Systematic review and meta-analysis. BMJ.

[4] Bokslag A., van Weissenbruch M., Mol B.W., de Groot C.J.M. (2016). Preeclampsia; short and long-term consequences for mother and neonate. Early Hum. Dev..

[5] Rana S., Lemoine E., Granger J.P., Karumanchi S.A. (2019). Preeclampsia: Pathophysiology, challenges, and perspectives. Circ. Res..

[6] O’Hara M.W., Wisner K.L. (2014). Perinatal mental illness: Definition, description and aetiology. Best. Pract. Res. Clin. Obstet. Gynaecol..

[7] Woody C.A., Ferrari A.J., Siskind D.J., Whiteford H.A., Harris M.G. (2017). A systematic review and meta-regression of the prevalence and incidence of perinatal depression. J. Affect. Disord..

[8] Stein A., Pearson R.M., Goodman S.H., Rapa E., Rahman A., McCallum M., Howard L.M., Pariante C.M. (2014). Effects of perinatal mental disorders on the fetus and child. Lancet.

[9] Slomian J., Honvo G., Emonts P., Reginster J.Y., Bruyère O. (2019). Consequences of maternal postpartum depression: A systematic review of maternal and infant outcomes. Womens Health.

[10] Gjerdingen D.K., Yawn B.P. (2007). Postpartum depression screening: Importance, methods, barriers, and recommendations for practice. J. Am. Board. Fam. Med..

[11] Silverman M.E., Reichenberg A., Savitz D.A., Cnattingius S., Lichtenstein P., Hultman C.M., Larsson H., Sandin S. (2017). The risk factors for postpartum depression: A population-based study. Depress. Anxiety.

[12] Yildiz P.D., Ayers S., Phillips L. (2017). The prevalence of posttraumatic stress disorder in pregnancy and after birth: A systematic review and meta-analysis. J. Affect. Disord..

[13] Blom E.A., Jansen P.W., Verhulst F.C., Hofman A., Raat H., Jaddoe V.W., Coolman M., Steegers E.A.P., Tiemeier H. (2010). Perinatal complications increase the risk of postpartum depression. The Generation R Study. BJOG.

[14] Sep S., Verbeek J., Koek G., Smits L., Spaanderman M., Peeters L. (2010). Clinical differences between early-onset HELLP syndrome and early-onset preeclampsia during pregnancy and at least 6 months postpartum. Am. J. Obstet. Gynecol..

[15] Dowlati Y., Herrmann N., Swardfager W., Liu H., Sham L., Reim E.K., Lanctôt K.L. (2010). A meta-analysis of cytokines in major depression. Biol. Psychiatry.

[16] Miller A.H., Maletic V., Raison C.L. (2009). Inflammation and its discontents: The role of cytokines in the pathophysiology of major depression. Biol. Psychiatry.

[17] Miller G.E., Chen E., Zhou E.S. (2007). If it goes up, must it come down? Chronic stress and the hypothalamic–pituitary–adrenocortical axis in humans. Psychol. Bull..

[18] Bloch M., Schmidt P.J., Danaceau M., Murphy J., Nieman L., Rubinow D.R. (2000). Effects of gonadal steroids in women with a history of postpartum depression. Am. J. Psychiatry.

[19] Satapathy P., Gaidhane A.M., Vadia N., Menon S.V., Chennakesavulu K., Panigrahi R., Bushi G., Singh M., Sah S., Turkar A. (2025). Exposure to violence and risk of hypertensive disorders in pregnancy: Systematic review and meta-analysis. Eur. J. Obstet. Gynecol. Reprod. Biol. X.

[20] Yim I.S., Tanner Stapleton L.R., Guardino C.M., Hahn-Holbrook J., Dunkel Schetter C. (2015). Biological and psychosocial predictors of postpartum depression: Systematic review and call for integration. Annu. Rev. Clin. Psychol..

[21] Lahti-Pulkkinen M., Girchenko P., Tuovinen S., Sammallahti S., Reynolds R., Lahti J., Heinonen K., Lipsanen J., Hamalainen E., Villa P.M. (2020). Maternal Hypertensive Pregnancy Disorders and Mental Disorders in Children. Hypertension.

[22] Ye Y., Chen L., Xu J., Dai Q., Luo X., Shan N., Qi H. (2021). Preeclampsia and Its Complications Exacerbate Development of Postpartum Depression: A Retrospective Cohort Study. Biomed. Res. Int..

[23] Cox J.L., Holden J., Sagovsky R. (1987). Detection of postnatal depression. Development of the 10-item Edinburgh Postnatal Depression Scale. Br. J. Psychiatry.

[24] Wallis A., Fernandez R., Oprescu F., Cherecheş R., Zlati A., Dungy C. (2012). Validation of a Romanian scale to detect antenatal depression. Open Med..

[25] Levis B., Negeri Z., Sun Y., Benedetti A., Thombs B.D. (2020). DEPRESsion Screening Data (DEPRESSD) EPDS Group. Accuracy of the Edinburgh Postnatal Depression Scale (EPDS) for screening to detect major depression among pregnant and postpartum women: Systematic review and meta-analysis of individual participant data. BMJ.

[26] Mbarak B., Kilewo C., Kuganda S., Sunguya B.F. (2019). Postpartum depression among women with pre-eclampsia and eclampsia in Tanzania; a call for integrative intervention. BMC Pregnancy Childbirth.

[27] Shang J., Hackett M.L., Harris K., Woodward M., Roberts L.M., Zhang P., Henry A. (2024). Mental health in the two years following hypertensive and normotensive pregnancy: The Postpartum, Physiology, Psychology and Paediatric follow-up (P4) cohort study. Pregnancy Hypertens..

[28] Soehl J.R., Anthony K., Matovina C.N., Ward L.G., Stroud L.R., Miller E.S. (2024). Association between antepartum admission and postpartum depressive symptoms. Am. J. Obstet. Gynecol. MFM.

[29] Burton G.J., Redman C.W., Roberts J.M., Moffett A. (2019). Pre-eclampsia: Pathophysiology and clinical implications. BMJ.

[30] Morris G., Walder K., Berk M., Carvalho A.F., Marx W., Bortolasci C.C., Yung A.R., Puri B.K., Maes M. (2022). Intertwined associations between oxidative and nitrosative stress and endocannabinoid system pathways: Relevance for neuropsychiatric disorders. Prog. Neuropsychopharmacol. Biol. Psychiatry.

[31] Trifu S., Vladuti A., Popescu A. (2019). The neuroendocrinological aspects of pregnancy and postpartum depression. Acta Endocrinol..

[32] American College of Obstetricians and Gynecologists (ACOG) Screening for Perinatal Mental Health. Practice Bulletin No. 238, 2023. https://www.acog.org/clinical/clinical-guidance/clinical-practice-guideline/articles/2023/06/screening-and-diagnosis-of-mental-health-conditions-during-pregnancy-and-postpartum.

[33] Hoedjes M., Berks D., Vogel I., Franx A., Bangma M., Darlington A.S., Visser W., Duvekot J.J., Habbema J.D., Steegers E.A. (2011). Postpartum depression after mild and severe preeclampsia. J. Womens Health.

[34] Liu C.H., Zhang H.Y., Wang F., Mu S.S., Wen F.Y. (2025). Anxiety and hypertensive disorders of pregnancy: Epidemiology, mechanisms, and management strategies. World J. Psychiatry.

[35] Caropreso L., de Azevedo Cardoso T., Eltayebani M., Frey B.N. (2020). Preeclampsia as a risk factor for postpartum depression and psychosis: A systematic review and meta-analysis. Arch. Womens Ment. Health.

[36] Roberts L., Henry A., Harvey S.B., Homer C.S.E., Davis G.K. (2022). Depression, anxiety and posttraumatic stress disorder six months following preeclampsia and normotensive pregnancy: A P4 study. BMC Pregnancy Childbirth.

